# A Continuous Microfluidic Concentrator for High-Sensitivity Detection of Bacteria in Water Sources

**DOI:** 10.3390/mi13071093

**Published:** 2022-07-10

**Authors:** Seunghee Choo, Hyunjung Lim, Tae Eun Kim, Jion Park, Kyu Been Park, Chaewon Park, Chae Seung Lim, Jeonghun Nam

**Affiliations:** 1 College of Life Sciences and Bio Engineering, Incheon National University, Incheon 22012, Korea; tmdgl2887@gmail.com; 2 Artificial Intelligence (AI)-Bio Research Center, Incheon Jaeneung University, Incheon 21987, Korea; hyunjunglim.email@gmail.com (H.L.); kh0313k@gmail.com (T.E.K.); 3 Department of Song-do Bio Engineering, Incheon Jaeneung University, Incheon 21987, Korea; 96jion21@gmail.com; 4 Department of Song-do Bio Life Engineering, Incheon Jaeneung University, Incheon 21987, Korea; pkb0607sg@gmail.com (K.B.P.); pcw7732@naver.com (C.P.); 5 Department of Laboratory Medicine, College of Medicine, Korea University, Seoul 08307, Korea

**Keywords:** water contamination, bacteria, concentration, viscoelastic fluid

## Abstract

Water contamination is a critical issue that threatens global public health. To enable the rapid and precise monitoring of pathogen contamination in drinking water, a concentration technique for bacterial cells is required to address the limitations of current detection methods, including the culture method and polymerase chain reaction. Here we present a viscoelastic microfluidic device for the continuous concentration of bacterial cells. To validate the device performance for cell concentration, the flow characteristics of 2-μm particles were estimated in viscoelastic fluids at different concentrations and flow rates. Based on the particle flow distributions, the flow rate factor, which is defined as the ratio of the inlet flow rate to the outlet flow rate at the center outlet, was optimized to achieve highly concentrated bacterial cells by removal of the additional suspending medium. The flow characteristics of 0.5-, 0.7-, and 1.0-μm-diameter particles were evaluated to consider the effect of a wide spectrum of bacterial size distribution. Finally, the concentration factor of bacterial cells, Staphylococcus aureus, suspended in a 2000-ppm polyethylene oxide solution was found to be 20.6-fold at a flow rate of 20 μL/min and a flow rate factor of 40.

## 1. Introduction

Water contamination is a global challenge that restricts the amount of drinkable water. According to the World Health Organization (WHO), more than 2.6 billion people lack access to safe drinking water, and 3.4 million people, mostly children, die annually of water-related diseases. Contamination of drinking water by pathogenic bacteria is a major cause of water quality impairment worldwide. Pathogenic bacteria that contaminate water resources include *Escherichia coli*, *Salmonella*, *Pseudomonas aeruginosa*, *fecal coliform bacteria*, *Yersinia enterocolitica*, and *Cryptosporidium oocysts*.

*Staphylococcus aureus* (*S. aureus*), a Gram-positive rod-shaped bacterium, is a major pathogenic organism in drinking water and food [1]. There have been reports of *S. aureus*–induced infections in several water resources [2,3,4]. *S. aureus* can cause skin infections, pseudomembranous enteritis, endocarditis, bloodstream infections, sepsis, enterocolitis, osteomyelitis, and pneumonitis [5,6,7,8,9]. 

The conventional method for pathogen detection and identification is the microbial culture method [10]. Briefly, the water sample is filtered through a membrane filter (0.45 μm pore; Millipore, Billerica, MA, U.S.A.). The membranes are transferred to agar plates and cultured for 1–3 days. This gold standard method is precise; however, it is time-consuming for bacterial growth and requires multiple pieces of equipment and complicated analytical operations. To address the limitations of the current culture method, various techniques, including a polymerase chain reaction (PCR), loop-mediated isothermal amplification (LAMP), recombinase polymerase amplification (RPA), and an enzyme-linked immunosorbent assay (ELISA) have been developed as alternatives for bacterial detection [11,12,13], but they have not yet completely replaced the culture method due to the need for skilled operators, expensive equipment, and a low bacterial count [14]. Therefore, the bacterial concentration as a sample pretreatment is essential for the rapid and accurate detection of bacteria in water resources.

As a sample preparation process, centrifugation can be conducted for the bacterial cell concentration. However, because of the small size of the bacteria, the centrifugation process is labor-intensive, time-consuming, and less portable for a point-of-care (POC). In addition, centrifugal damage can alter the surface properties and interior structures of bacterial cells [15]. According to recent advancements in microfluidic technologies, microfluidics can be a potential solution for sample preparation tools for a POC by shortening the processing time and reducing possible damage to cells.

Among the various microfluidic techniques, viscoelastic non-Newtonian microfluidics has gained much attention owing to the intrinsic nonlinear elastic forces in polymer solutions [16,17]. It enables the easier manipulation of cells without complex channel structures compared to previous microfluidic techniques that do not use external forces, such as inertial microfluidics [18,19]. In addition, viscoelastic cell manipulation can be achieved over a wide range of flow rates by simply modulating the rheological properties of the fluid and flow rates. In a viscoelastic non-Newtonian fluid, a nonuniform distribution of the first normal stress difference (*N*_1_) can drive suspended cells laterally in a straight rectangular channel. Therefore, it has been applied in focusing [17,20,21] and separating [22,23,24,25,26] particles/cells depending on the size in a continuous flow. In addition, a non-electrically powered continuous cell concentration device suitable for on-site usage has recently been developed [27]. To the best of the authors’ knowledge, it has not been applied to a continuous concentration of bacterial cells as a sample preparation tool for rapid and accurate water quality assessment. 

Here, we demonstrate a viscoelastic microfluidic device for the continuous concentration of bacterial cells. We examined the flow characteristics of the particles at different polymer solution concentrations and flow rates. The wide size distribution of the bacterial cells was determined by examining the concentration performance of particles with different sizes (0.5, 0.7, 1.0, and 2.0 μm). Finally, bacterial cells, *S. aureus*, were used to verify the device’s performance for bacteria concentration. After sample processing, bacterial cells were concentrated and analyzed using flow cytometry and real-time LAMP (RT-LAMP).

## 2. Materials and Methods

### 2.1. Device Fabrication

A PDMS microchannel was fabricated using a soft lithography technique with a replica mold, which was fabricated using an SU-8-negative photoresist (MicroChem, Newton, MA, USA) patterned on a silicon wafer. The PDMS base and curing agent (Sylgard 184; Dow Corning, Midland, MI, USA) were mixed at a ratio of 10:1, degassed in a vacuum chamber, and thermally cured in an oven for 1 h at 80 °C. The cured PDMS channels were peeled from the replica mold and bonded to a glass slide with oxygen plasma (CUTE; Femto Science, Seoul, Korea). 

**Figure 1:**
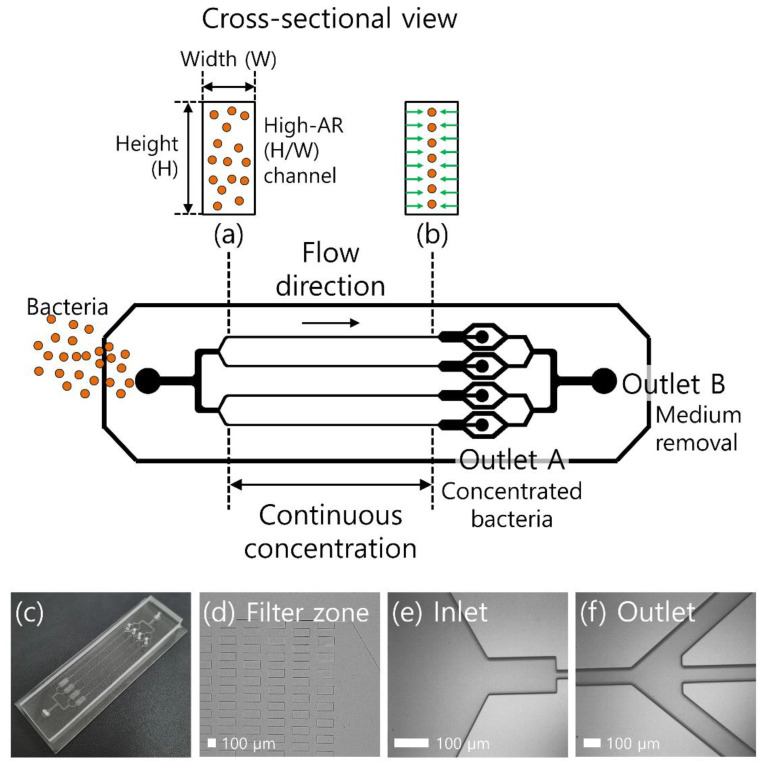
Schematic of the continuous bacterial concentration using viscoelastic fluid. (**a**) Bacteria suspended in a viscoelastic fluid are randomly introduced to the inlet. (**b**) Due to the elastic force, bacteria cells are focused at the center of the microchannel. At the outlet, tightly focused cells are collected at the center outlet (outlet A) and suspending medium is removed to the side outlets (outlet B). (**c**) Image of the fabricated device used in this study for bacterial concentration. Microscopic images of (**d**) the filter zone, (**e**) the inlet, and (**f**) the outlet region of the microchannel.

The microfluidic device consists of four parallel microchannels with one inlet and two outlets. For a single channel, the width, height, and length of the main straight microchannel were 20 μm, 75 μm, and 3 cm, respectively, while the widths of the outlet trifurcation channels were 150, 100, and 150 μm. To prevent the nonuniform flow behaviors among four parallel microchannels induced by dust or debris in each channel, the microfluidic filter structures were designed in the inlet region (Figure 1d).

### 2.2. Sample Preparation

A viscoelastic non-Newtonian fluid (1000-, 2000-, and 3000-ppm PEO; Mw 600 kDa) was prepared in PBS as a suspending medium. The viscosity and the relaxation time of the 1000-ppm PEO solution were 1.41 mPa·s and 0.55 ms, respectively [28]. Fluorescent polystyrene particles with diameters of 0.5, 0.7, 1.0, and 2.0 μm were used to validate the device performance as an analog to the bacterial cells. The particles were suspended in 1000-, 2000-, and 3000-ppm PEO solutions at approximately 1 × 10^5^ particles/mL.

For biological application, *S. aureus* was used. Frozen stock cultures of strain were maintained at −80 °C [29]. Before the microfluidic experiments, the staphylococci were cultivated overnight in Mueller–Hinton broth with 2% sodium chloride and washed thrice with PBS. For the final application of bacterial concentration, the bacterial cells were stained using propidium iodide (PI; Sigma Aldrich, St. Louis, MO, USA) for visualization, spiked in deionized water (DI water) to a concentration of 3 × 10^3^/mL, and mixed with 4000-ppm PEO solution at a ratio of 1:1. The final concentration of bacterial cells was 1.5 × 10^3^/mL in the 2000-ppm PEO solution. 

### 2.3. Experimental Procedure and Post Analysis

The sample solution flow rate was controlled using a syringe pump (LSP01-1A Longer Precision Pump). During the experiment, fluorescent particles and bacterial cells were monitored using an inverted microscope (CKX41; Olympus, Tokyo, Japan) equipped with a fluorescent camera (CS230B; Olympus, Tokyo, Japan). 

A flow cytometry analysis was conducted to evaluate device performance using a flow cytometer (Navios EX; Beckman Coulter, Inc., CA, USA). The inlet sample and each sample from the two outlets were incubated with PI in the dark to label and quantitatively analyze the bacteria. Ten microliters of the sample were added to 490 μL of PBS containing fluorescent beads at a known number (TruCOUNT; BD Ltd., Oxford, UK).

The DNA was extracted using a bacterial DNA kit (Genolution Inc., Seoul, Korea) following the manufacturer’s instructions. Briefly, the sample was heated with lysis solution (BD Ltd., Oxford, UK) at 100 °C for 5 min. The sample was loaded into the extraction plate, and the extraction process was done through the equipment (Genolution Inc., Seoul, Korea). LAMP was performed using a DNA LAMP kit (M monitor, Daegu, Korea). The sequences of the LAMP primers targeting the 16S rRNA gene of S. aureus were as follows: F3 (TGGAATTCCATGTGTAGCGG), B3 (AGGCGGAGTGCTTAATTGC), FIP (TCGCACATCAGCGTCAGTTACA-ATGCGCAGAGATATGGAGGA), BIP (AGATACCCTGGTAGTCCACGCC-CACTAAGGGGCGGAAACC), LF (CCAGAAAGTCGCCTTCGCCACT), and LB (AAACCATGAGTGCTAAGTGTTAGG). The LAMP reactive mixture contained 12.5 μL of reaction mixture, 2 μL of enzyme mixture, 3.25 μL of distilled water, 1.25 μL of primer set, 1 μL of Eva green (25×), and 5 μL of template DNA to form a total volume of 25 μL. The composition of the LAMP primer set included 4 μM of two outer primers (F3 and B3), 32 μM of two inner primers (FIP and BIP), and 10 μM of loop primers (LF and LB). The LAMP assay was run on the CFX 96 Touch Real-Time PCR Detection System (Bio-Rad Laboratories, Hercules, CA, USA) at 64 °C for 60 min.

## 3. Results

A schematic representation of the proposed device for the bacteria concentration in a viscoelastic non-Newtonian fluid is shown in Figure 1. The device consists of four parallel channels connected to one inlet and two outlets [30], because viscoelastic focusing can be achieved without introducing sheath fluids. The initial sample mixture contained bacterial cells suspended in a viscoelastic fluid. At the inlet, bacterial cells were randomly injected into the microchannel, as shown in Figure 1a. When viscoelastic fluid is injected into the microchannel, the elastic force (*F_e_*) affects the center plane of the microchannel owing to the nonuniform first normal stress difference [17,31,32]. In addition, the inertial lift force affects the lateral migration of cells in viscoelastic fluids as follows:

(1) $$F_{e}\sim a^{3}\frac{\partial N_{1}}{\partial x}\sim \lambda {\left(a/W\right)}^{3}Q^{3}$$

(2) $$F_{i}=F_{i,s}+F_{i,w}\sim \rho {\left(a/W\right)}^{4}Q^{2}$$

where *x*, *N*_1_, *λ*, *a*, *W*, *Q*, and ρ are the lateral distance, first normal stress difference, relaxation time, cell diameter, microchannel width, flow rate, and solution density, respectively. Cells suspended in a viscoelastic fluid are affected by the synergistic effect of fluid elasticity and inertia, which focus the bacterial cells toward the center of the high-aspect-ratio (AR) channel (Figure 1b). Tightly focused cells can be collected at the center outlet (outlet A), whereas additional suspending medium can be removed via the side outlets (outlet B). Therefore, the bacteria collected at the central outlet are highly concentrated.

To characterize the flow of the viscoelastic fluid, nondimensional numbers are adopted, such as the Reynolds number (*Re*), Weissenberg number (*Wi*), and elasticity number (*El*). Re is defined as the ratio of the inertial force to the viscous force and Wi is the ratio of the elastic force to the viscous force. *El* shows the relative effect of fluid elasticity on inertia:

(3) $$Re=\frac{\rho V_{m}D_{h}}{\eta }$$

(4) $$Wi=\lambda \dot{\gamma_{c}}$$

(5) $$El=\frac{Wi}{Re}$$

where *V_m_*, *D_h_*, *η*, and $\dot{\gamma_{c}}$ indicate the mean flow velocity, hydraulic diameter of the particle, characteristic viscosity of the solution, and characteristic shear rate, respectively.

**Figure 2:**
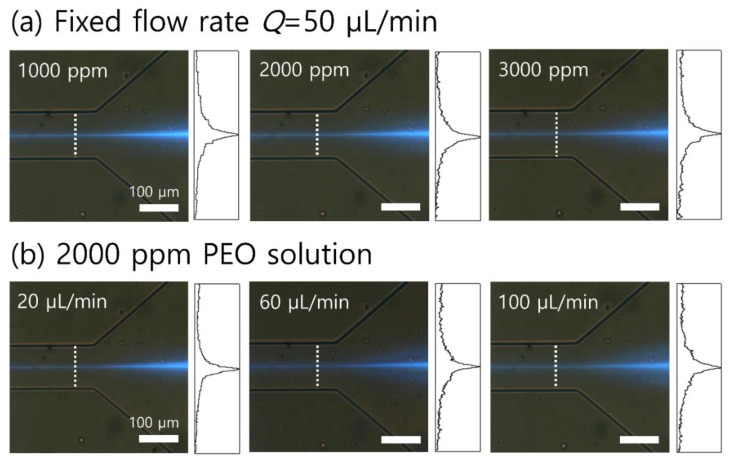
Evaluation of (**a**) the effect of the polymer concentration (1000, 2000, and 3000 ppm) at the fixed flow rate of 50 μL/min and (**b**) the effect of the flow rate (20, 60, and 100 μL/min) in the 2000-ppm PEO solution on flow characteristics of 2-μm fluorescent polystyrene particles. White dotted lines show the position of fluorescent intensity measurement. The X and Y axes indicate the normalized fluorescence intensity of 0–1 and channel width of 0–100 μm, respectively.

To examine the effect of viscoelasticity and flow rates on the flow characteristics of the 2-μm fluorescent polystyrene particles (blockage ratio β~0.1), the distributions of particles suspended in polyethylene oxide (PEO) solutions were observed. Figure 2a shows the fluorescent images and normalized fluorescence intensities in the expansion region at a flow rate of 50 μL/min using particles suspended in 1000-, 2000-, and 3000-ppm PEO solutions, respectively. The width of the focused 2-μm particles was determined by the region where the normalized fluorescence intensity was higher than 0.3. In the 1000-ppm PEO solution (*Re* = 3.76, *Wi* = 7.63, *El* = 2.03), 2-μm particles were focused along the channel center within the region of approximately 12.2% of the total width. As the concentration of the PEO solution increased, the focused widths of the 2-μm particles became wider. In the 2000- and 3000-ppm PEO solutions, the 2-μm particles were focused within ~14.7% and ~16.2% of the total width near the channel centerline. In the 1000-, 2000-, and 3000-ppm PEO solutions, the 2-μm particles (*β =* 0.1) showed a similar lateral migration displacement toward the centerline of the channel, which seemed to be saturated [22]. Considering the wide range of bacterial cells and moderate range of elasticity, a 2000-ppm PEO solution was used for further experiments. In the 1000-ppm PEO solution, bacterial cells with *β* < 0.1 might not be fully focused at the channel center due to decreased elasticity. Meanwhile, the increased contribution of elasticity in the 3000-ppm PEO solution might not be comparable to inertia, that is, *El >* O (1), which limits the synergistic effect of inertia and elasticity [24]. The flow rate could not be increased for the 3000-ppm PEO solution considering the flow resistance in the microchannel due to the deformation of the polydimethylsiloxane (PDMS) channel. Using a rigid, deformation-free plastic-based microfluidic device with leak-tight connections [33], the device throughput and concentration performance can be further enhanced, finally enabling the commercialization process.

The flow rate dependence of the 2-μm particle distribution in the 2000-ppm PEO solution was examined at various flow rates of 20, 60, and 100 μL/min, as shown in Figure 2b. The focused widths of the 2-μm particles were 14.3, 14.8, and 16.0% at 20, 60, and 100 μL/min, respectively. With an increased flow rate, *Re* and *Wi* increased; however, *El* remained nearly constant and the flow characteristics of the particles were dependent on the flow rate [24]. A tight focusing of the 2-μm particles for a high concentration factor was attainable at a low flow rate of 20 μL/min.

During the concentration process, the suction flow rate at outlet A could be controlled to enhance the concentration of the 2-μm particles at outlet A. The flow rate factor (FF) is defined as the ratio of the inlet flow rate to the outlet flow rate at the target outlet, outlet A [34,35]. The FF was recently modulated in the viscoelastic flow to achieve high-efficiency separation and concentration of Candida cells [35], which have different hydraulic diameter ranges than bacteria.

**Figure 3:**
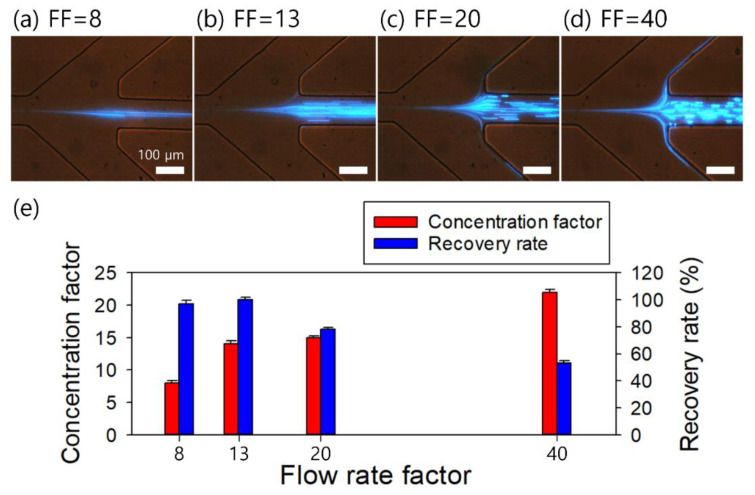
Evaluation of the effect of the flow rate factors (FF) determined by the suction flow rate from outlet A on the flow characteristics of the 2-μm fluorescent particles in 2000-ppm PEO solution. (**a**) FF = 8, (**b**) FF = 13, (**c**) FF = 20, (**d**) FF = 40. (**e**) Recovery rate and concentration factor at various FF values. The standard deviations depict the measured values from five different experiments (*n* = 5).

The initial FF of the device, four, was determined by the widths of the outlet channels designed as 150, 100, and 150 μm, respectively. The effect of the suction flow rate on the concentration performance was evaluated with a controlled suction flow rate at outlet A, using a syringe pump (KDS210; KD Scientific, Holliston, MA, USA) (Figure 3). As shown in Figure 3a–d, center-focused 2-μm particles flowed to outlet A at a suction flow rate of 2.5 μL/min (FF = 8). When the FF was increased to 13 (suction flow rate at outlet A = 1.5 μL/min), almost all of the 2-μm particles were still collected at outlet A. However, when the FF was increased to 20 (suction flow rate at outlet A = 19 μL/min), a few of the center-focused particles could not be recovered at outlet A. As the FF was further increased to 40 (suction flow rate at outlet A = 19.5 μL/min), many of the 2-μm particles deflected to the side outlets.

Figure 3e shows the FF-dependent concentration factor and recovery rate. The concentration factor is defined as the ratio of the particle concentration collected at outlet A to the initial particle concentration at the inlet, whereas the recovery rate is defined as the ratio of the number of particles collected at outlet A to the total number of particles in the collected sample. The number of particles in each sample was counted using a hemocytometer. The recovery rate persisted to exceed 97% when the FF increased to 13. However, as it increased further (FF = 20 and 40), the recovery rates decreased to 78% and 53% because a certain number of particles was deflected to outlet B. On the other hand, the concentration factor continued to increase from 8 to 22 with an increased FF due to the removal of additional suspending medium from outlet B. At an FF of 66.6 (suction flow rate at outlet B = 19.8 μL/min), the concentration factor and recovery rate could not be evaluated because the 2-μm particles at the center outlet started to flow back to the trifurcation region and flowed to outlet B. Therefore, with the simultaneous consideration of the concentration factor and recovery rate, the optimal FF was 40 in this study. With the determined flow condition (injection flow rate, 20 μL/min; FF = 40), the initial sample of 400 μL can be processed in our device in 20 min to achieve 10 μL of a concentrated output with an expected concentration factor of 40, which is sufficient for use in post-analysis such as PCR and fluorescence-activated cell sorting (FACS). For further optimization under the same flow conditions, viscoelastic fluids with different elasticities such as xanthan gum [36] can be selected, and the cross-sectional shape of the microchannel can be varied to a triangular or trapezoidal shape [37,38,39] to enhance the concentration factor or reduce the processing time.

**Figure 4:**
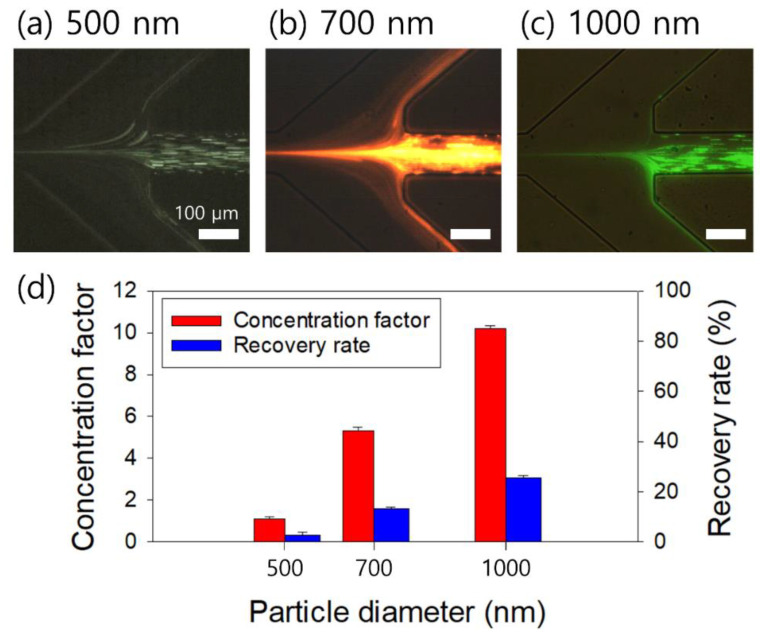
(**a**–**c**) Flow characteristics of nanoparticles with 500, 700, and 1000 nm diameters, suspended in 2000-ppm PEO solution at 20 μL/min and FF = 40. (**d**) Recovery rate and concentration factor of the nanoparticles. The standard deviations depict the measured values from five different experiments (*n* = 5).

Considering the wide size distribution of the S. aureus sample (0.5–2 μm) [40], the flow characteristics of nanoparticles with diameters of 500, 700, and 1000 nm were evaluated. The concentration (2000-ppm PEO solution), inlet flow rate (20 μL/min), and outlet suction flow rate (FF = 40) of the polymer solution were determined based on the results shown in Figure 2 and Figure 3. As shown in Figure 4a, 500-, 700-, and 1000-nm particles were focused near the centerline of the microchannel. However, unlike the 2-μm particles (β = 0.1), the 500-, 700-, and 1000-nm particles were not tightly focused at the microchannel center because of the small blockage ratios (β = 0.025, 0.035, 0.05), since both inertial and elastic forces are dependent on particle size [24]. Then, a large number of nanoparticles are deflected to the side outlets.

Figure 4b shows the particle size-dependent concentration factor and recovery rate evaluated at outlet A. With large-sized particles, the concentration factor and recovery rate increased. The recovery rates were 2.7%, 13.2%, and 25.4% and the concentration factors were 1.1, 5.3, and 10.2 for the 500-, 700-, and 1000-nm particles, respectively.

**Figure 5:**
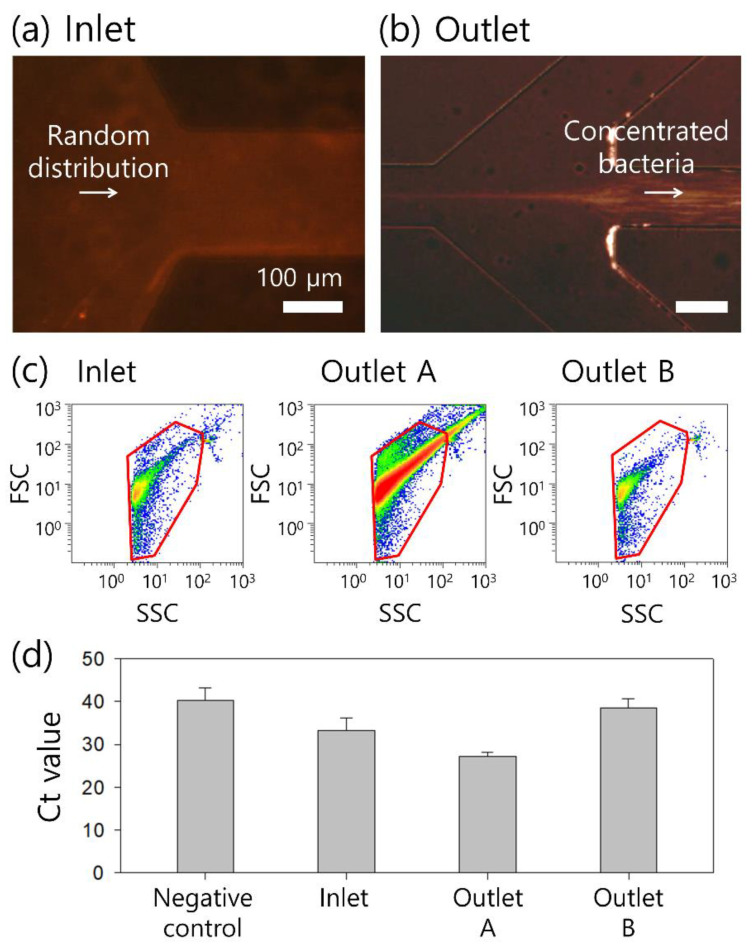
Concentration of bacteria cells at an injection flow rate of 20 μL/min and FF of 40 at the (**a**) inlet and (**b**) outlet after the concentration process. (**c**) Flow cytometric scattergrams before and after the concentration process at the inlet, outlet A, and outlet B, respectively. (**d**) Ct values of RT-LAMP assay before and after the concentration process.

*S. aureus* cells were used for the final application of the continuous concentration system. Figure 5a,b show microscopic images at the inlet and outlet of the microchannel during the concentration process at a fixed flow rate of 20 μL/min. Randomly injected bacterial cells were focused along the centerline of the microchannel after the viscoelastic concentration process, and cell-free additional buffer flowed out to the side outlets with an FF of 40. Therefore, the bacterial cells of interest were collected at outlet A.

To evaluate the concentration performance, a flow cytometric analysis was conducted before and after the concentration process, as shown in Figure 5c. The concentrations of fluorescent-dyed bacteria were 1.5 × 10^3^/mL at the inlet, 3.1 × 10^4^/mL at outlet A, and 7.4 × 10^2^/mL at outlet B. Based on the cytometric analysis, the concentration factors of the bacterial samples collected at outlets A and B were calculated. Focused bacterial cells collected at outlet A were concentrated 20.6-fold, while those at outlet B were diluted 0.5-fold due to the additional amount of medium. Meanwhile, the recovery rates of the bacterial cells at each outlet were 51.6% at outlet A and 48.1% at outlet B. The concentration factor and recovery rate of *S. aureus* cells with a wide size distribution of 0.5–2 μm exhibited smaller values than those of the 2-μm particles. Compared to fluorescent polystyrene particles, bacteria vary widely in size, shape, and arrangement; therefore, their concentration performance can differ from that of particles. Various studies on the flow characteristics according to the cell shape were recently reported [23]; however, few have examined a small size range such as bacteria. Therefore, for further studies, fundamental research is required to estimate the effect of the bacterial shape on the separation and concentration performance.

Quantitative RT-LAMP analyses were performed on the bacterial samples before and after the concentration process. As shown in Figure 5d, the Ct (Cycle threshold) value for the *S. aureus* sample at the inlet prior to concentration was 33.22, while that for the negative control sample was 40.22. After the concentration process, the Ct values for the samples collected at outlets A and B were 27.17 and 38.57, respectively. This was due to the concentration of bacterial cells after removal of the additional buffer solution at outlet B. Meanwhile, the effect of the viscoelastic fluid [2000 ppm PEO (600 kDa) solution] was examined by comparison of the LAMP analysis results of bacteria suspended in the viscoelastic fluid and phosphate-buffered saline (PBS) at the same concentrations. There was no significant effect of viscoelastic fluid on Ct values, 21.44 for the PBS-suspended sample at 10^6^/mL and 21.64 for the PEO-based sample at 10^6^/mL (data not shown).

## 4. Discussion

For further applications of our continuous concentration device, other bacterial cells with different physical properties in water resources will be considered. For example, *Salmonella*, *E. coli*, *P. aeruginosa*, and *Y. enterocolitica* are rod-shaped bacteria with lengths of 1–5 μm and widths of 0.25–1.5 μm [41,42,43,44]. Fungal contaminants can also be found in drinking water. The effect of the shape difference of the contaminants on the viscoelastic concentration must be examined [23]. 

Our viscoelastic device enables a continuous concentration for the pretreatment of rare pathogens. However, for our device to enhance water quality monitoring sensitivity, its throughput is required to be improved. Device throughput can be further enhanced by device stacking and increasing the number of parallel devices [45,46,47]. The aspect ratio of the microchannel can be further increased by fabricating the device in a rigid thermoplastic resin [48], which is suitable for mass production during commercialization. 

Meanwhile, bloodstream bacterial infections, a critical cause of severe sepsis, septic shock, and multiple organ failure syndrome with high morbidity and mortality rates, are also possible [49]. Our viscoelastic continuous concentration device has the potential for a further application of the sample preparation process for the clinical diagnosis of bacterial infections.

## 5. Conclusions

In summary, herein we developed a novel viscoelastic cell concentration device that enables the rapid detection of pathogen contamination in water resources. The effects of the polymer concentration of the viscoelastic fluid and the flow rate on the viscoelastic focusing of 2-μm particles were evaluated. Particles 2 μm in diameter were tightly focused at 20 μL/min in a 2000-ppm PEO solution. In addition, the suction flow rate at outlet A was controlled to achieve a maximum concentration factor of 22 and an FF of 40. For bacterial diagnosis, a wide size distribution of bacterial cells was considered by evaluating the flow characteristics of 500-, 700-, and 1000-nm fluorescent particles. Finally, *S. aureus* bacterial cells were concentrated with a concentration factor of 20.6 and a recovery rate of 51.6% on FACS and RT-LAMP. These results confirmed that the proposed viscoelastic concentration device had a considerably high concentration factor.
